# Acute Psychosis Related to Primary Hyperparathyroidism in a Patient With Bipolar Disorder

**DOI:** 10.7759/cureus.42567

**Published:** 2023-07-27

**Authors:** Zahid Khan, Gideon Mlawa, Hussameldin Mahdi, Mohammed Abumedian

**Affiliations:** 1 Acute Medicine, Mid and South Essex NHS Foundation Trust, Southend-on-Sea, GBR; 2 Cardiology, Bart’s Heart Centre, London, GBR; 3 Cardiology and General Medicine, Barking, Havering and Redbridge University Hospitals NHS Trust, London, GBR; 4 Cardiology, Royal Free Hospital, London, GBR; 5 Internal Medicine and Diabetes and Endocrinology, Barking, Havering and Redbridge University Hospitals NHS Trust, London, GBR; 6 Gastroenterology, Barking, Havering and Redbridge University Hospitals NHS Trust, London, GBR; 7 Geriatrics, Barking, Havering and Redbridge University Hospitals NHS Trust, London, GBR

**Keywords:** hyperparathyroidism-induced psychosis, hyperthyroidism-induced hypercalcemia, hypercalcemic psychosis, recurrent psychosis, adult primary hyperparathyroidism

## Abstract

Primary hyperparathyroidism (PHPT) can cause hypercalcemia secondary to high parathyroid hormone secretion. Hyperparathyroidism- and hypercalcemia-related acute psychotic symptoms can be challenging to diagnose in patients with mental health-related disorders, and it should be considered a possible differential in these patients besides medications. It can sometimes be the first manifestation of the disease, and diagnosis can be challenging, especially in patients with a previous psychiatric history without checking their biochemistry profile. The hypercalcemia severity can vary from mild to severe, and signs and symptoms may also vary depending on the calcium levels. Hypercalcemia can cause neuropsychiatric dysfunction, and patients may present with confusion, agitation, delusions, and hallucinations. We present a case of a 54-year-old patient with a previous history of bipolar disorder and a recent diagnosis of depression and schizophreniform disorder, who presented to the emergency department with acute agitation, violent behavior, and disorientation. She was being managed by the community mental health team at a local behavioral health hospital for new onset psychosis over the past few months. She was refusing blood tests prior to hospital admission. Calcium level on laboratory tests was 3.54 mmol/l, and parathyroid hormone level was 45 pg/ml. She was managed with intravenous fluids initially, followed by zoledronic acid (4 mg intravenously over 15 minutes). She was then commenced on cinacalcet 30 mg twice daily initially, which was later increased to 60 mg twice daily. Ultrasound of the neck demonstrated a large left parathyroid mass, and she underwent left parathyroidectomy as an urgent outpatient. She has remained asymptomatic, and her psychiatry symptoms resolved following parathyroidectomy.

## Introduction

Primary hyperparathyroidism (PHPT) is a condition in which the parathyroid glands produce extra parathyroid hormone resulting in elevated calcium levels greater than 2.6 mmol/L in the blood. This is usually caused by a solitary parathyroid adenoma, and the reported incidence is 21 cases per 100,000 person-years [1]. Hypercalcemia is a common clinical condition, and risk factors include primary hyperparathyroidism, various sporadic and genetic conditions, renal failure, and medications such as calcium, vitamin D supplements, vitamin A, thiazide diuretics, lithium, parenteral nutrition, theophylline, foscarnet, growth hormone, omeprazole, aromatase inhibitors, parathyroid hormone (PTH) analogs [2]. Patients suffering from PHPT may present with a complex set of skeletal, renal, and psychiatric symptoms; however, psychiatric symptoms are not considered an indication for surgery [3]. Hypercalcemia is more likely to cause neuropsychiatric symptoms including mood and cognitive changes and, very rarely, acute psychosis [4]. Although hypercalcemia-induced psychotic symptoms tend to resolve quickly with the normalization of calcium levels, symptoms of psychosis may not resolve in all these cases [1]. It has been suggested that hypercalcemia-related mood and cognition changes could be due to its role in the metabolism of monoamines in the central nervous system by modulating dopaminergic and cholinergic metabolism and neurotransmission along synaptic junctions [1].

The reported incidence of neuro-psychiatric symptoms is about 4.2% in patients with hypercalcemia [5]. These symptoms can be divided into three categories: confusional psychosis, characterized by a change in consciousness ranging from drowsiness to stupor, paranoid psychosis with clear sensorium and patients having severe depression and paranoid delusions, and pseudoneurotic form showing a history of complaints such as fatigue, lassitude, weakness, poor appetite, and constipation [3]. Mild to moderate hypercalcemia (serum calcium level: 2.5-3.49 mmol/L) is associated with depression, apathy, irritability, and lack of initiative and spontaneity, whereas severe hypercalcemia (serum calcium level > 3.5 mmol/L) is associated with psychosis, catatonia, and lethargy [1].

## Case presentation

A 54-year-old female patient presented to the hospital with agitation, delusions, hypervigilance, paranoia, and violent behavior over the past few weeks. She had a past medical history of bipolar disorder, diagnosed at the age of 31, depression at the age of 33, and schizophreniform disorder three months ago due to acute changes in her behavior including agitation, delusions, and anger issues. Her regular medications include sertraline 100 mg once daily, aripiprazole 15 mg once daily for over 15 years, and risperidone 2 mg twice daily, which was started about three months back due to her new symptoms. She was managed for acute psychosis over the past three months by a psychiatrist at a local psychiatric hospital, and the nursing staff noticed worsening behavioral symptoms such as somatic delusions, confusion, agitation, and occasionally violent behavior over the last three to four weeks. The somatic delusional symptoms included the patient's belief in malfunctioning of both upper and lower limbs, and the catatonic symptoms included echolalia, negativism, sudden agitation, and refusal to eat and drink for the past three to four weeks. She was hemodynamically stable, and the Glasgow coma scale was 14 (E4M6V4) due to confusion. She was admitted to the hospital, and she initially refused all investigations including blood tests and permitted only limited physical examination which was normal. She consented to blood tests after 12 hours, and laboratory tests showed a high calcium level of 3.54 mmol/L. She was commenced on intravenous normal saline and received about three liters of normal saline over 24 hours. Repeat blood tests showed only mild improvement in hypercalcemia from 3.54 to 3.25 mmol/L, and the PTH level was reported as 45 pg/ml (Table 1).

**Table 1:** Serial lab values

Blood test	Day 1	Day 2	Day 5	Day 7	6 months	Reference range
Hemoglobin	14	13.5	13	12.5	13	12-16 g/dL
Platelet	300	307	315	314	325	150-400 × 10^9^/L
Urea	3.2	3.5	2.8	3.6	4.0	1.8-7.1 mmol/L
Creatinine	65	60	55	65	58	53-97 µmol/L
Sodium	136	138	137	138	140	135-145 mmol/L
Potassium	4.6	4.5	4.0	4.2	4.5	3.6-5.2 mmol/L
Magnesium	0.92	0.88	-	0.85	-	0.85-1.10 mmol/L
Adjusted calcium	3.54	3.25	2.95	2.55	2.42	2.1-2.6 mmol/L
Phosphate (mmol/L)	0.69	0.59	0.58	0.48	0.95	0.97-1.45 mmol/L
Parathyroid hormone level	45	-	-	-	35	10-55 pg/mL
Total vitamin D	39	-	-	-	55	>50 nmol/L
Serum folate	3.9	-	-	-	3.6	2.7-17.0 ng/mL

**Figure 1:**
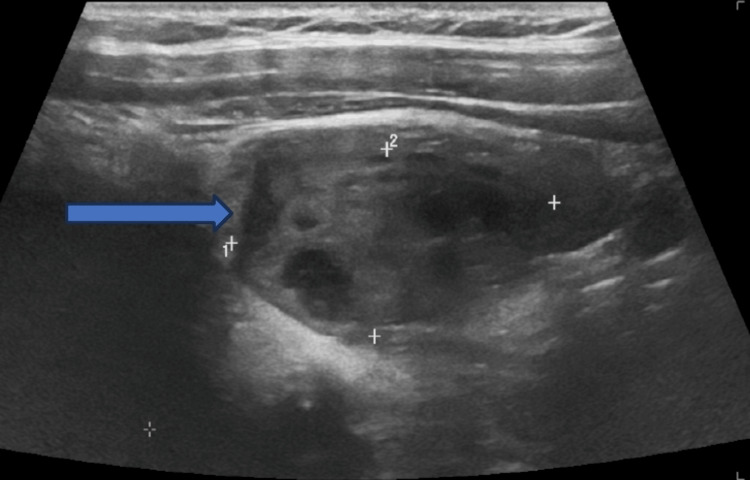
Ultrasound of the neck shows parathyroid adenoma as shown by the pointed arrow

**Figure 2:**
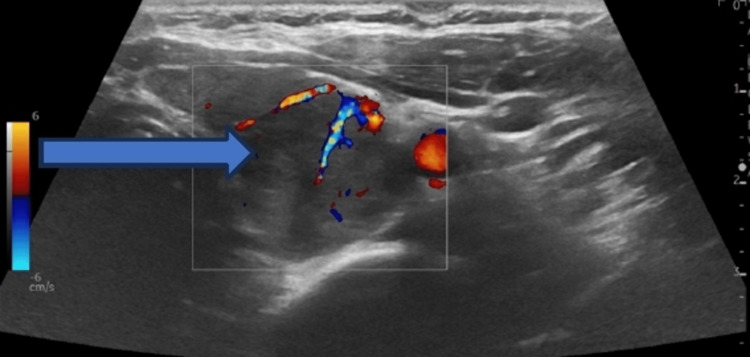
Color Doppler ultrasound scan shows parathyroid adenoma (pointed arrow)

**Figure 3:**
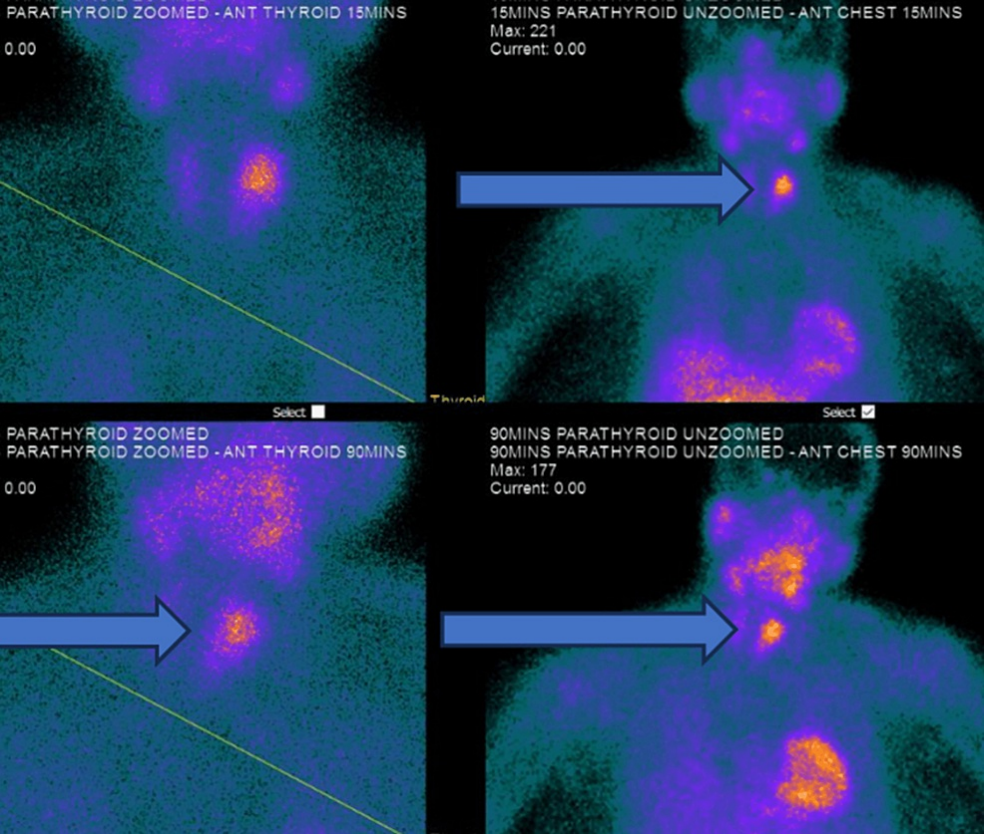
Technetium-99m-sestamibi scintigraphy shows intense uptake in the left parathyroid gland (pointed arrows)

A computerized scan of the head did not reveal any structural brain abnormality. The arterial blood gas was unremarkable. She was administered intravenous zoledronic acid 4 mg intravenously and continued to receive intravenous normal saline infusion. She had a review by the endocrinology and diabetes team that advised her to continue with intravenous fluids and commence on cinacalcet 30 mg twice daily prior to discharge. Ultrasound of the neck showed a large left parathyroid gland mass suggestive of left parathyroid adenoma with a few small cystic components and color blood flow demonstrated internally (Figures 1, 2). The patient also underwent technetium-99m-sestamibi scintigraphy that demonstrated intense tracer uptake in the left parathyroid gland (Figure 3).

She was also reviewed by the mental health team who advised her to hold her regular medications including risperidone and aripiprazole. She continued to receive intravenous fluids for four to five days, and her calcium level improved to 2.55 mmol/L after five days. The patient’s confusion level improved, and she was not showing any violent behavior anymore. She also had magnetic resonance imaging of the head (MRI), which was normal. She was discharged back to the behavior health unit on cinacalcet 30 mg twice daily initially, which was then increased to 60 mg twice daily. The patient was referred to the surgical team for outpatient parathyroidectomy, and the calcium level normalized following parathyroidectomy. She has remained stable from a mental health perspective and was discharged from the endocrine clinic after a year follow-up. Regular medications including sertraline and aripiprazole were restarted, and risperidone was permanently stopped.

## Discussion

The key differential diagnosis in this patient was a psychotic disorder, schizoaffective disorder, schizophrenia, bipolar II disorder, bipolar I disorder with psychotic features, and any other medical pathology contributing to these symptoms. Patients with hypercalcemia can present with a variety of psychiatric sequelae that ranges from anxiety and mood disorders to cognitive disorders [2]. Hyperparathyroidism is being detected and diagnosed more frequently with the advent of newer imaging techniques and laboratory tests [6]. Primary hyperparathyroidism is more common in women, with a reported prevalence of 4/1000 women aged over 60 years [7]. Most patients with hypercalcemia are asymptomatic, and symptomatic patients mostly have bone pains and deformities, fractures, arthralgia, urinary signs, confusion, fatigue, and increased thirst [6,8]. Parathyroid adenoma is the most common cause of primary hyperparathyroidism, and ectopic locations include intrathyroidal location, lateral-cervical, paraaortic position, para esophageal, retropharyngeal, anterior mediastinum, and aortopulmonary flow [6,9,10]. Patients with suspected parathyroid adenoma should have a cervical ultrasound scan, and in the case of ectopic parathyroid adenoma, a technetium 99-sestamibi scan is a more sensitive diagnostic test [8].

The severity of psychotic symptoms is usually dependent on the degree of hypercalcemia; however, this correlation is inconclusive in patients with PHPT [11,12]. One study reported that even mild hypercalcemia can cause significant symptoms if they have a history of hypertension, old age, white matter lesions on computed tomography scans, recent bereavements, and underlying cognitive impairment [13]. The exact mechanism by which PHPT causes psychotic symptoms remains unclear; however, calcium has been identified to have a role in the monoamine metabolism in the central nervous system through the modulation of dopaminergic and cholinergic metabolism. Similarly, increased calcium level has been observed in schizophrenia patients during catatonic episodes [6].

About 30% of patients with PHPT-related hypercalcemia are asymptomatic and are accidentally diagnosed with routine blood tests. Besides physical examination including neck examination, further investigations should include ECG to look for shortened QT interval, J-waves, and ventricular arrhythmias in extremely low or high calcium levels. Blood tests could be useful to differentiate between primary and tertiary hyperparathyroidism, which is common in patients with chronic kidney disease. Urinalysis may demonstrate calciuresis in patients with low serum calcium levels to help identify patients with familial hypocalciuric hypercalcemia [5].

Previous studies have shown a poor correlation between the serum calcium level and the severity of psychotic symptoms [2,5,6]. Chiba et al. reported a serious psychotic episode in a patient with mild hypercalcemia, which resolved with parathyroidectomy [5]. The prevalence of psychotic symptoms was found to be 5.2% in elderly Singaporean patients based on the Well-being of the Singapore Elderly (WiSE) study [14]. A study in the United Kingdom reported the prevalence of schizophrenia as 0.1%-0.5% as compared to a 1% lifetime prevalence [15]. Hypercalcemia should be considered a possible differential diagnosis in these patients. Several randomized controlled trials (RCT) have reported varied results with regard to the resolution of psychiatric symptoms and parathyroidectomy. Few RCTs showed improvement in symptoms, whereas a meta-analysis demonstrated no benefits of parathyroidectomy in patients with neuropsychiatric symptoms [16-18]. Our patient showed complete resolution of symptoms following parathyroidectomy.

Lithium-induced hyperparathyroidism-related hypercalcemia is a rare complication of lithium in patients with bipolar disorder; however, she never received lithium therapy. These patients usually have the classic signs and symptoms of “bones (osteoporosis), stones (renal calculi), thrones (constipation), abdominal groans (pancreatitis), and psychiatric overtones (delirium).” The hypercalcemia-related symptoms mostly resolve by stopping lithium; however, some patients may require parathyroidectomy [19].

## Conclusions

Hypercalcemia is an important cause of acute psychosis in patients. Patients with underlying psychiatric history can present a diagnostic challenge, and clinicians should consider this a possible differential diagnosis. It is vital to consider acute medical problems when facing such challenging cases due to the fact that biochemical abnormalities can contribute to worsening symptoms. Most patients with primary hyperparathyroidism-related hypercalcemia show complete resolution of symptoms following curative parathyroidectomy. The long-term prognosis is favorable in patients undergoing parathyroidectomy for primary hyperparathyroidism. The exact mechanism by which hypercalcemia leads to acute psychotic symptoms remains unelucidated.

## References

[1] Parks KA, Parks CG, Onwuameze OE, Shrestha S (2017). Psychiatric complications of primary hyperparathyroidism and mild hypercalcemia. Am J Psychiatry.

[2] Meng AZ, Tan Y, Ong SJ, Wee BB, Teo L (2022). Primary hyperparathyroidism causing psychosis: a case report. Cureus.

[3] Nagy L, Mangini P, Schroen C, Aziz R, Tobia A (2020). Prolonged hypercalcemia-induced psychosis. Case Rep Psychiatry.

[4] Bojdani E, Zhang T, Shetty S, Tahera D (2018). Hypercalcemia and psychosis: case report, review of the literature, and management considerations. Prim Care Companion CNS Disord.

[5] Chiba Y, Satoh K, Ueda S, Kanazawa N, Tamura Y, Horiuchi T (2007). Marked improvement of psychiatric symptoms after parathyroidectomy in elderly primary hyperparathyroidism. Endocr J.

[6] Otsuki K, Izuhara M, Miura S (2021). Psychosis in a primary hyperparathyroidism patient with mild hypercalcemia: a case report. Medicine (Baltimore).

[7] Soto-Pedre E, Newey PJ, Leese GP (2023). Stable incidence and increasing prevalence of primary hyperparathyroidism in a population-based study in Scotland [IN PRESS]. J Clin Endocrinol Metab.

[8] Silaghi H, Valea A, Ghervan C, Silaghi AC (2011). Ectopic intrathyroid parathyroid adenoma: diagnostic and therapeutic challenges due to multiple osteolytic lesions. Case report. Med Ultrason.

[9] Karvounaris DC, Symeonidis N, Triantafyllou A, Flaris N, Sakadamis A (2010). Ectopic parathyroid adenoma located inside the hypoglossal nerve. Head Neck.

[10] Yadav R, Mohammed TL, Neumann DR, Mihaljevic T, Hoschar A (2010). Case of the season: ectopic parathyroid adenoma in the pericardium: a report of robotically assisted minimally invasive parathyroidectomy. Semin Roentgenol.

[11] Henry JF, Sebag F, Tamagnini P, Forman C, Silaghi H (2004). Endoscopic parathyroid surgery: results of 365 consecutive procedures. World J Surg.

[12] Papa A, Bononi F, Sciubba S, Ursella S, Gentiloni-Silveri N (2003). Primary hyperparathyroidism: acute paranoid psychosis. Am J Emerg Med.

[13] Silverberg SJ, Walker MD, Bilezikian JP (2013). Asymptomatic primary hyperparathyroidism. J Clin Densitom.

[14] Singh P, Bauernfreund Y, Arya P, Singh E, Shute J (2018). Primary hyperparathyroidism presenting as acute psychosis secondary to hypercalcaemia requiring curative parathyroidectomy. J Surg Case Rep.

[15] McDonald CA, Bruce DG, Smith DJ (2002). Primary hyperparathyroidism in an elderly woman: surgical reversibility of profound mental state problems due to mild hypercalcaemia. Int J Geriatr Psychiatry.

[16] Talpos GB, Bone HG 3rd, Kleerekoper M (2000). Randomized trial of parathyroidectomy in mild asymptomatic primary hyperparathyroidism: patient description and effects on the SF-36 health survey. Surgery.

[17] Rao DS, Phillips ER, Divine GW, Talpos GB (2004). Randomized controlled clinical trial of surgery versus no surgery in patients with mild asymptomatic primary hyperparathyroidism. J Clin Endocrinol Metab.

[18] Ospina NS, Maraka S, Rodriguez-Gutierrez R (2016). Comparative efficacy of parathyroidectomy and active surveillance in patients with mild primary hyperparathyroidism: a systematic review and meta-analysis. Osteoporos Int.

[19] Naramala S, Dalal H, Adapa S, Hassan A, Konala VM (2019). Lithium-induced hyperparathyroidism and hypercalcemia. Cureus.

